# Supplementary material for: Drug targeting Nsp1-ribosomal complex shows antiviral activity against SARS-CoV-2
Source: eLife. 2022 Mar 24;11:e74877. doi: 10.7554/eLife.74877 (PMC9018067; doi:10.7554/eLife.74877)
Supplement: Supplementary file 1. [file elife-74877-supp1.docx]

**Supplementary File 1**

|  | Drug | Docking score | Clash score |
| --- | --- | --- | --- |
| 1 | Verapmil | 6.91 | 65 |
| 2 | Dopexamine | 6.76 | 68 |
| 3 | Lidoflazine | 6.52 | 70 |
| 4 | Doripenem | 6.42 | 58 |
| 5 | Bosentan | 5.80 | 102 |
| 6 | Montelukast | 5.61 | 138 |
| 7 | Saquinavir | 5.59 | 195 |
| 8 | Formoterol | 5.13 | 62 |
| 9 | Cloxacillin | 5.01 | 114 |
| 10 | Nicardipin | 4.98 | 89 |
| 11 | Perindoprin | 4.89 | 102 |
| 12 | Mupirocine | 4.67 | 118 |
