# Supplementary material for: Drug targeting Nsp1-ribosomal complex shows antiviral activity against SARS-CoV-2
Source: eLife. 2022 Mar 24;11:e74877. doi: 10.7554/eLife.74877 (PMC9018067; doi:10.7554/eLife.74877)
Supplement: Supplementary file 2. [file elife-74877-supp2.docx]

**Supplementary File 2**

| Primers | Sequence |
| --- | --- |
| *FLuc*_Fp | 5’-CAACTGCATAAGGCTATGAAGAGA-3’ |
| *FLuc* _Rp | 5’-ATTTGTATTCAGCCCATATCGTTT-3’ |
| GAPDH_Fp | 5’-ACAACTTTGGTATCGTGGAAGG-3’ |
| GAPDH_Rp | 5’-GCCATCACGCCACAGTTTC-3’ |
| Nsp1_Fp | 5’-GGGAATTCCATATGGAGAGCCTTGTCCCTGGTTTCAACG-3’ |
| Nsp1_Rp | 5’-CGCGGATCCTTACCCTCCGTTAAGCTCACGCATGAGTTC-3’ |
| Nsp1∆C_Rp | 5’- CGCGGATCCTTAATCAGTGCCAAGCTCGTCGCCT-3’ |
| SARS CoV-2 N-1_Fp | 5'GACCCCAAAATCAGCGAAAT3' |
| SARS CoV-2 N-1_Rp | 5' TCTGGTTACTGCCAGTTGAATCTG3', |
| Probe (6-FAM / BHQ-1) | 5-ACCCCGCATTACGTTTGGTGGACC-3’ |
