# Supplementary figures and images for: Drug targeting Nsp1-ribosomal complex shows antiviral activity against SARS-CoV-2
Source: eLife. 2022 Mar 24;11:e74877. doi: 10.7554/eLife.74877 (PMC9018067; doi:10.7554/eLife.74877)

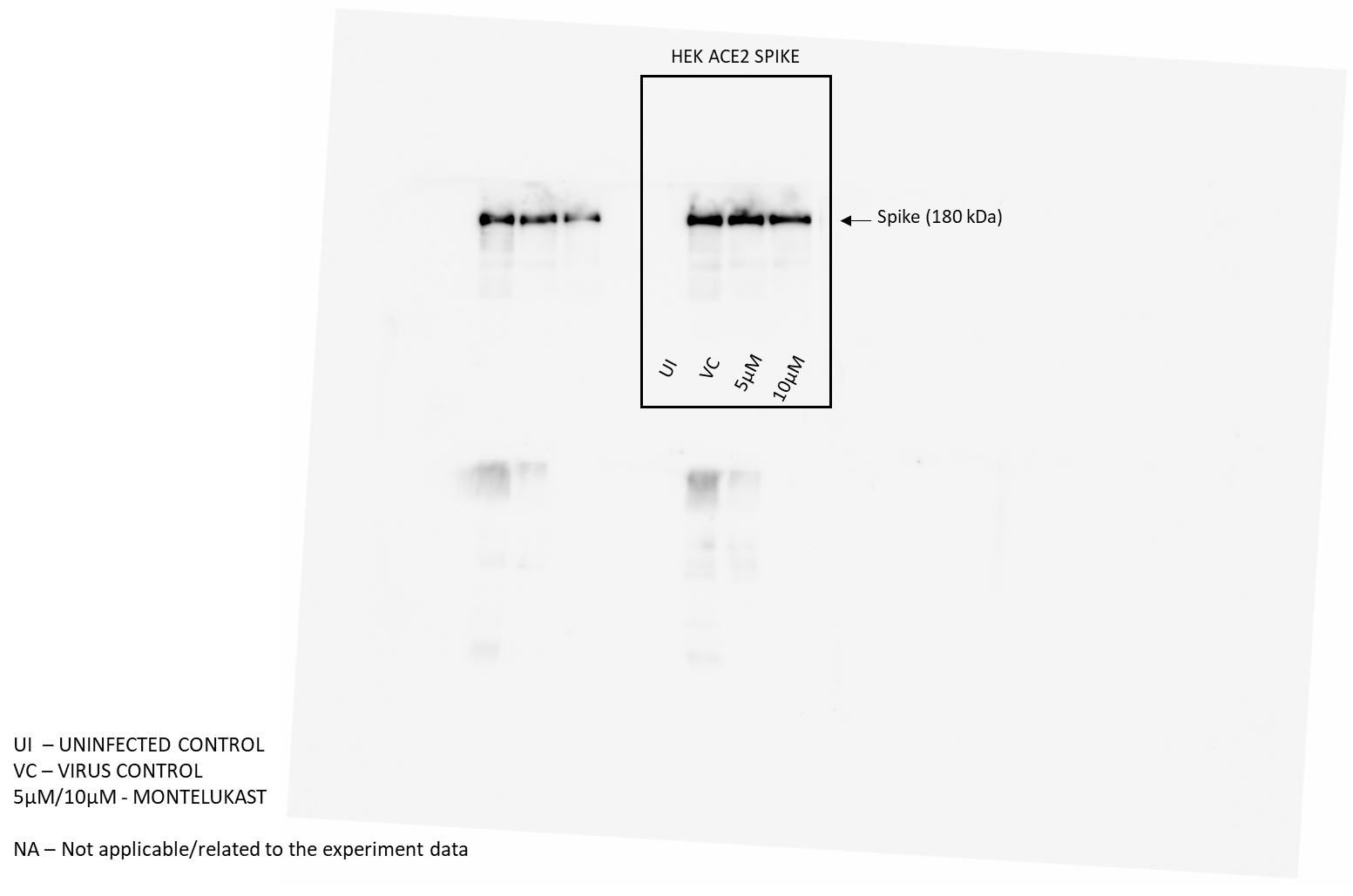

Supplement: Source data 1. [file elife-74877-data1.zip › Source data/Figure 3-supplement 2 A_Source data_1/HEK ACE Spike Saqunavir complete blot.tif.png]

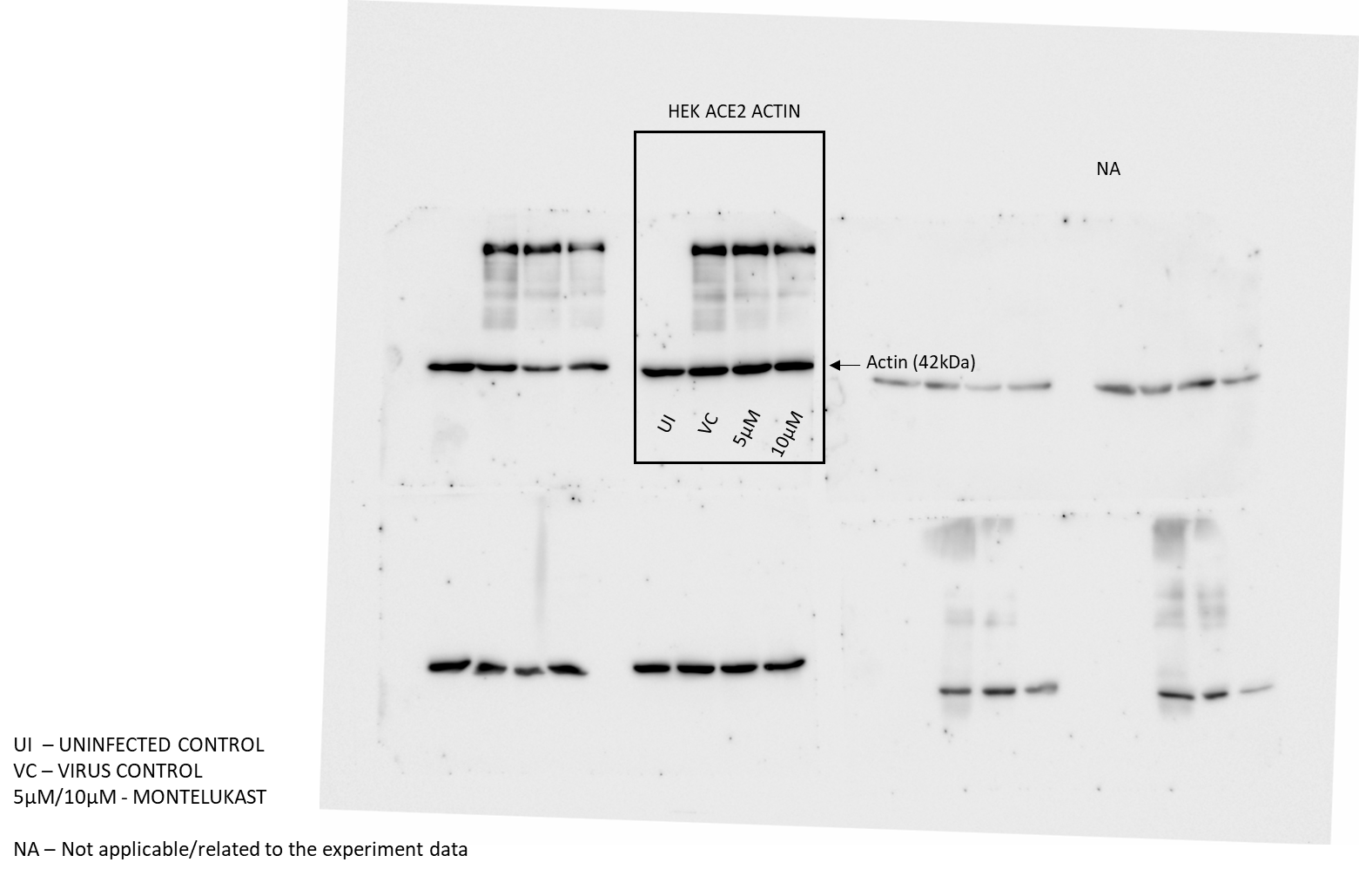

Supplement: Source data 1. [file elife-74877-data1.zip › Source data/Figure 3-supplement 2 A_Source data_1/HEK ACE2 Actin complete blot.png]

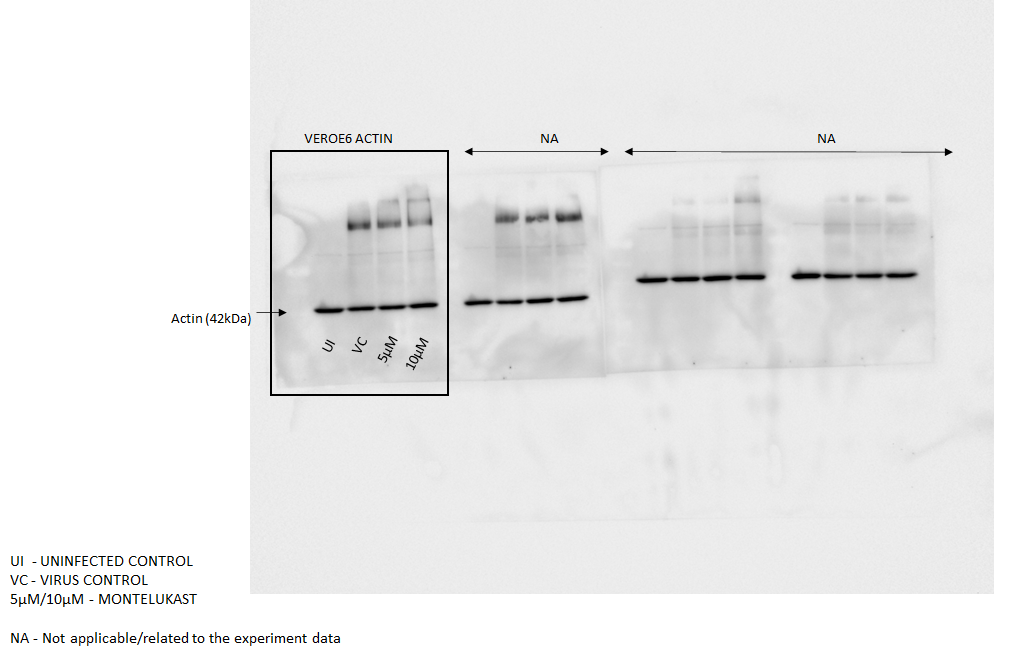

Supplement: Source data 1. [file elife-74877-data1.zip › Source data/Figure 3 D Source data 1/Vero actin complete blot.png]

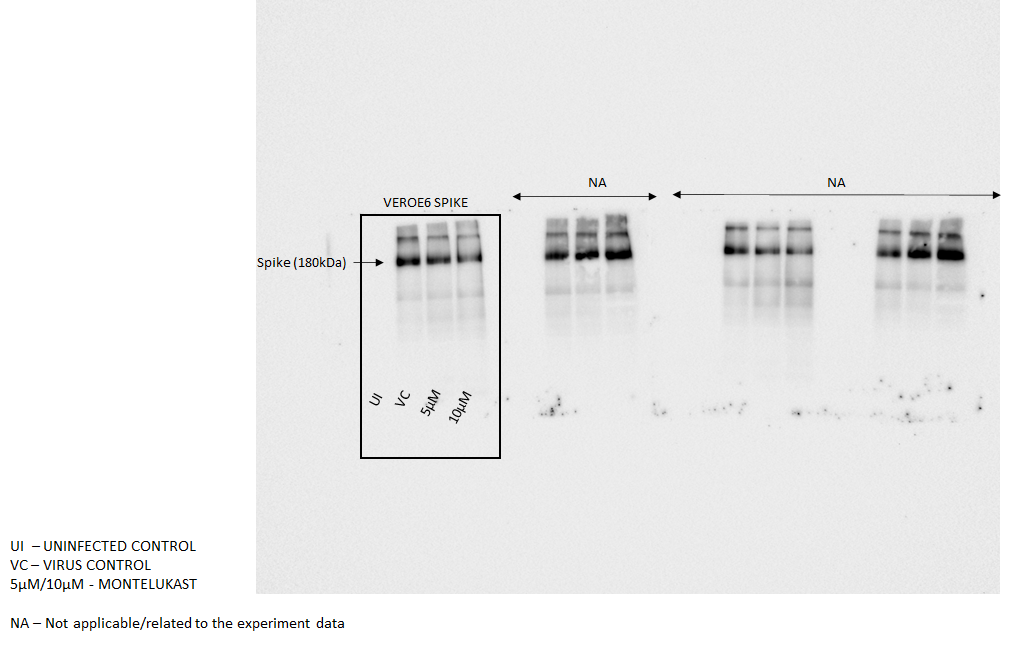

Supplement: Source data 1. [file elife-74877-data1.zip › Source data/Figure 3 D Source data 1/Vero Spike Montelukast complete blot.png]

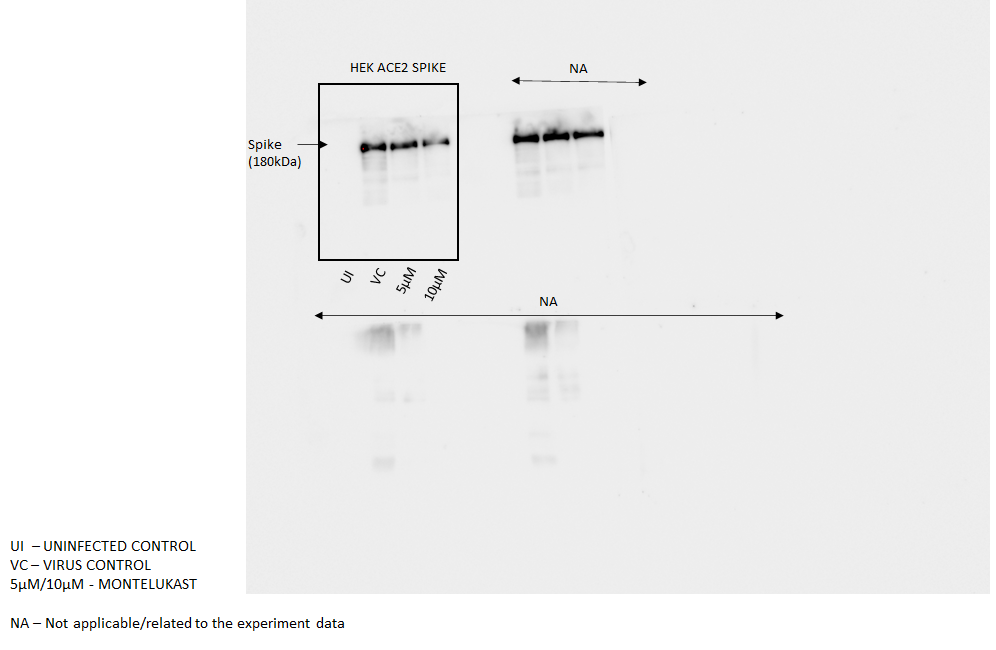

Supplement: Source data 1. [file elife-74877-data1.zip › Source data/Figure 3 A Source data 1/HEK ACE2 Spike Montelukast complete blot.png]

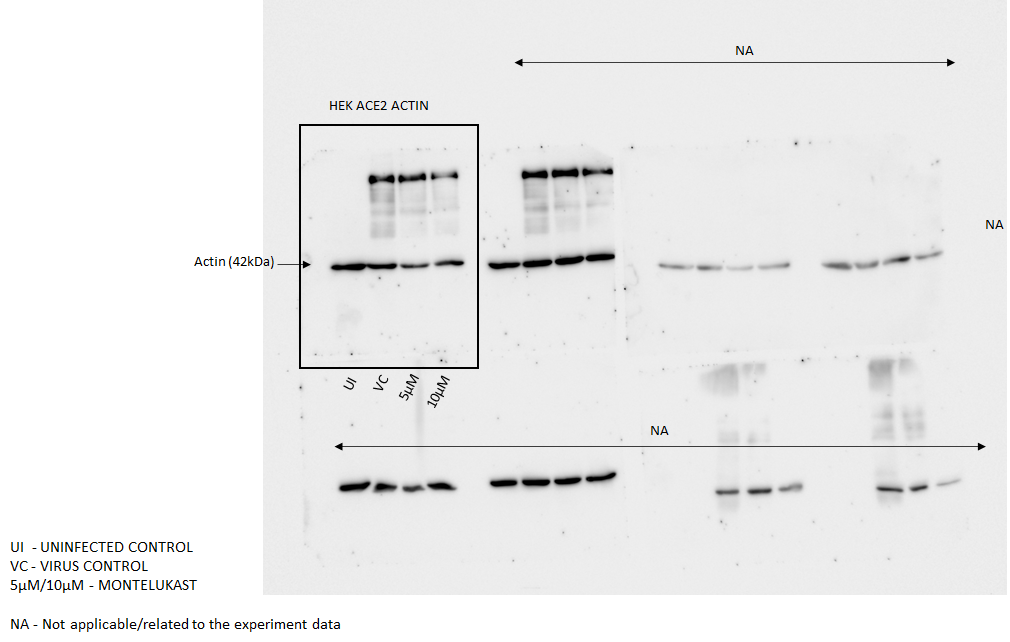

Supplement: Source data 1. [file elife-74877-data1.zip › Source data/Figure 3 A Source data 1/HEK ACE2 Actin complete blot.png]

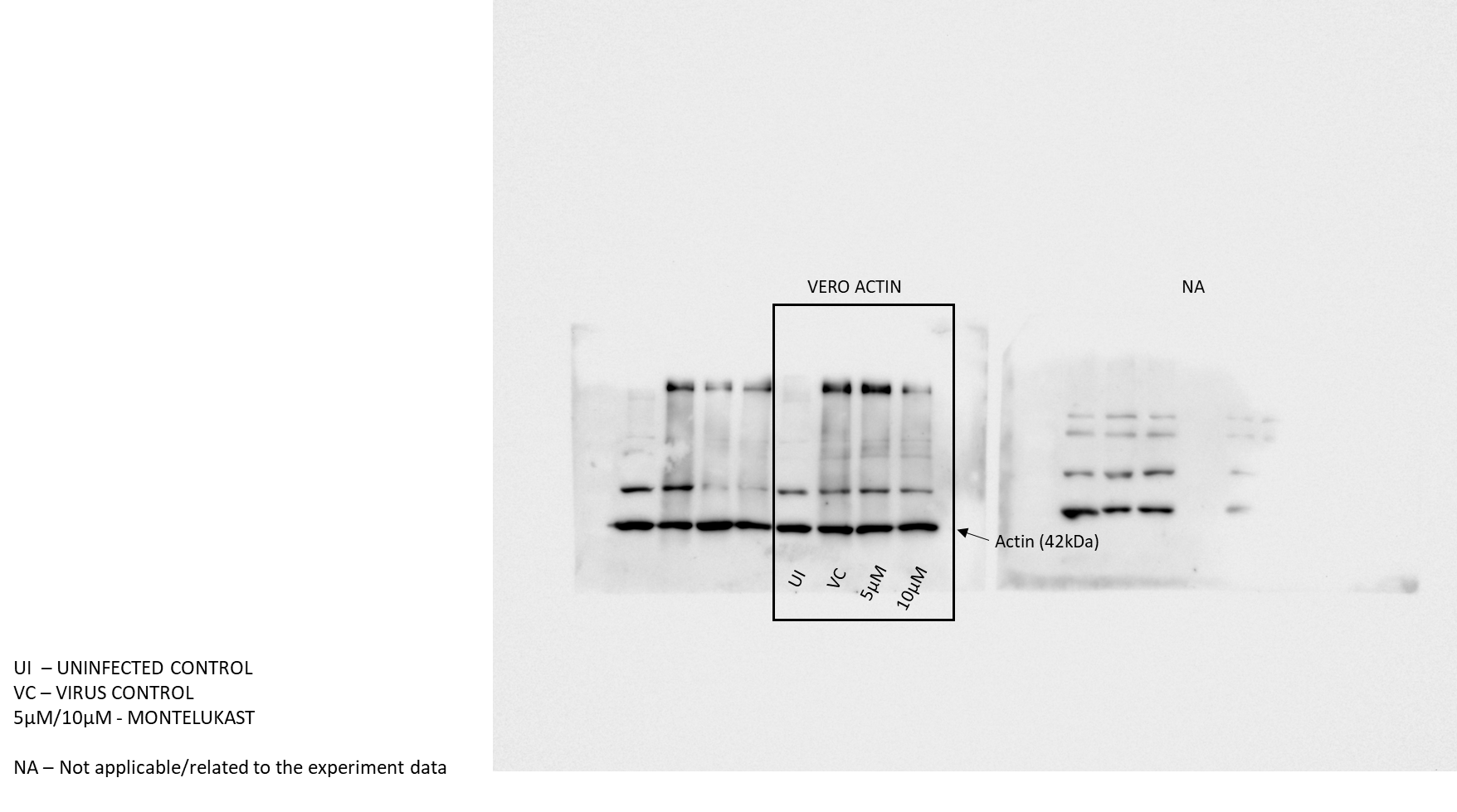

Supplement: Source data 1. [file elife-74877-data1.zip › Source data/Figure 3-supplement 2 D_Source data_1/Vero actin complete blot.png]

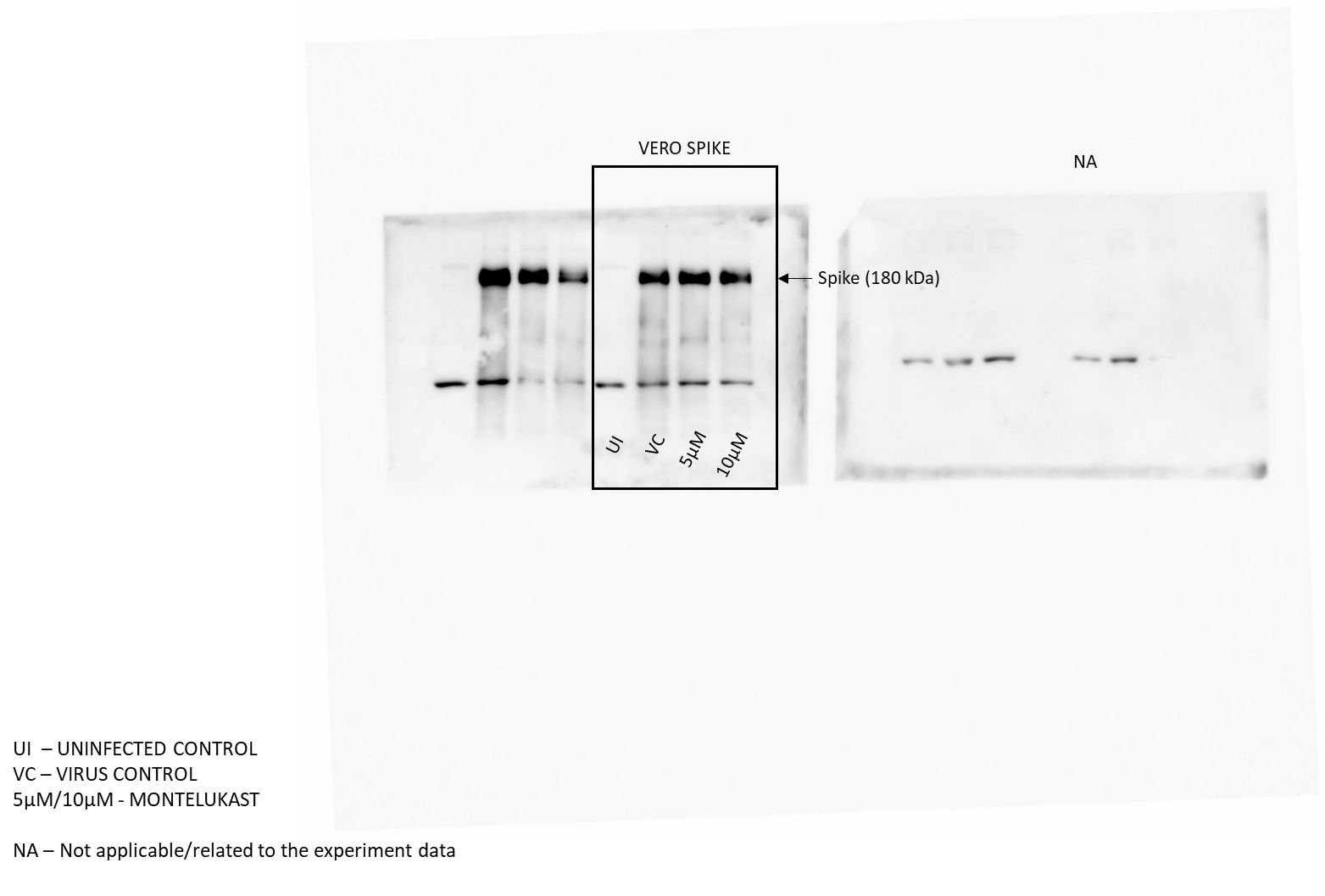

Supplement: Source data 1. [file elife-74877-data1.zip › Source data/Figure 3-supplement 2 D_Source data_1/Vero Spike Saquinavir complete blot.png]
