# Supplementary material for: Drug targeting Nsp1-ribosomal complex shows antiviral activity against SARS-CoV-2
Source: eLife. 2022 Mar 24;11:e74877. doi: 10.7554/eLife.74877 (PMC9018067; doi:10.7554/eLife.74877)
Supplement: Transparent reporting form [file elife-74877-transrepform1.docx]

***eLife’s* transparent reporting form**

We encourage authors to provide detailed information *within their submission* to facilitate the interpretation and replication of experiments. Authors can upload supporting documentation to indicate the use of appropriate reporting guidelines for health-related research (see [EQUATOR Network](http://www.equator-network.org/%20)), life science research (see the [BioSharing Information Resource](https://biosharing.org/)), or the [ARRIVE guidelines](http://www.plosbiology.org/article/info:doi/10.1371/journal.pbio.1000412) for reporting work involving animal research. Where applicable, authors should refer to any relevant reporting standards documents in this form.

If you have any questions, please consult our Journal Policies and/or contact us: [editorial@elifesciences.org](mailto:editorial@elifesciences.org).

**Sample-size estimation**

- You should state whether an appropriate sample size was computed when the study was being designed
- You should state the statistical method of sample size computation and any required assumptions
- If no explicit power analysis was used, you should describe how you decided what sample (replicate) size (number) to use

Please outline where this information can be found within the submission (e.g., sections or figure legends), or explain why this information doesn’t apply to your submission:

Not applicable: We have not included any clinical experiments in the report.

**Replicates**

- You should report how often each experiment was performed
- You should include a definition of biological versus technical replication
- The data obtained should be provided and sufficient information should be provided to indicate the number of independent biological and/or technical replicates
- If you encountered any outliers, you should describe how these were handled
- Criteria for exclusion/inclusion of data should be clearly stated
- High-throughput sequence data should be uploaded before submission, with a private link for reviewers provided (these are available from both GEO and ArrayExpress)

Please outline where this information can be found within the submission (e.g., sections or figure legends), or explain why this information doesn’t apply to your submission:

**For Molecular dynamic studies**: We have performed three independent replicates for each protein-ligand complex in Gromacs 2020.5 for 500ns and subsequent RMSD, RMSF and coordinates were extracted and plotted and presented manuscript. For binding energy calculation we have performed two independent replicates in Gromacs 2020.5 for 500ns and then subsequently subjected to gmx_MMPBSA for energy calculations. (More detailed information is available in materials and methods under the subheading of Receptor preparation for *in silico* studies and molecular screening of FDA-approved drugs)

**For Bio layer interferometry**: We have performed three independent experiments and data. The data was smoothened by Savistzky-Golay filtering at default settings. The kinetics properties were analyzed for Association and Dissociation in 1:1 binding mode by using the global fitting. (More detailed information is available in materials and methods under the subheading of Bio-layer Interferometry)

**For Nanoscale Differential Scanning Fluorometry:** We have performed three independent experiments and the representative experiment is shown in manuscript. The change in melting temperature is calculated by subtracting the Tm value of protein alone from protein-ligand complex. (More detailed information is available in materials and methods under the subheading of Nanoscale Differential Scanning Fluorometry).

**Real time PCR**: The results include data from one experiment containing three technical replicates of samples analyzed. One-way ANOVA with Dunnett’s multiple comparison test was used for statistical analysis.

**Plaque Assay:** Results include data from one experiment containing two technical replicates for each test sample analyzed. One-way ANOVA with Dunnett’s multiple comparison test was used for statistical analysis.

**Western blots**: The western blot images and corresponding quantifications of bands represent data from two independent biological experiments performed.

**Toxicity assay**: Results represent data from one experiment with three technical replicates of samples. One-way ANOVA with Dunnett’s multiple comparison test was used for statistical analysis.

**Translational rescue experiments**: We have performed these experiments in

triplicate manner (biological replicates) two times and one of the representative

experiments is shown in the manuscript. We have performed these experiments both in the presence of drugs montelukast and saquinavir at different concentrations independently. The cells were lysed 24 hrs post-transfection, and luciferase activity was measured. (More detailed information is available in materials and methods under the subheading of translational inhibition and rescue experiments.

**Statistical reporting**

- Statistical analysis methods should be described and justified
- Raw data should be presented in figures whenever informative to do so (typically when N per group is less than 10)
- For each experiment, you should identify the statistical tests used, exact values of N, definitions of center, methods of multiple test correction, and dispersion and precision measures (e.g., mean, median, SD, SEM, confidence intervals; and, for the major substantive results, a measure of effect size (e.g., Pearson's r, Cohen's d)
- Report exact p-values wherever possible alongside the summary statistics and 95% confidence intervals. These should be reported for all key questions and not only when the p-value is less than 0.05.

Please outline where this information can be found within the submission (e.g., sections or figure legends), or explain why this information doesn’t apply to your submission:

To evaluate the significance of the variation we applied unpaired t-test through assuming Gaussian distribution parametric test. The respective p-values are mentioned in figure legends.

(For large datasets, or papers with a very large number of statistical tests, you may upload a single table file with tests, Ns, etc., with reference to sections in the manuscript.)

**Group allocation**

- Indicate how samples were allocated into experimental groups (in the case of clinical studies, please specify allocation to treatment method); if randomization was used, please also state if restricted randomization was applied
- Indicate if masking was used during group allocation, data collection and/or data analysis

Please outline where this information can be found within the submission (e.g., sections or figure legends), or explain why this information doesn’t apply to your submission:

Not applicable: we have not performed animal studies.

**Additional data files (“source data”)**

- We encourage you to upload relevant additional data files, such as numerical data that are represented as a graph in a figure, or as a summary table
- Where provided, these should be in the most useful format, and they can be uploaded as “Source data” files linked to a main figure or table
- Include model definition files including the full list of parameters used
- Include code used for data analysis (e.g., R, MatLab)
- Avoid stating that data files are “available upon request”

Please indicate the figures or tables for which source data files have been provided:

Data for Molecular simulation dynamics, BLI, Nano-DSF, RT-PCR, Translational rescue experiments and binding energy calculations are provided as mentioned below:

**Figure 1-source data 1**: BLI screening

**Figure 1-source data 2**: BLI Kinetics

**Figure 1-source data 3**: Nano-DSF

**Figure 1-source data 4**: RMSD + Binding energy

**Figure 2-source data**: Translational rescue and RT-PCR

**Figure 3 A-source data 1**: Western blot (Spike protein expression in the presence of Montelukast in HEK-ACE2 cells).

**Figure 3 D-source data 1**: Western blot (Spike protein expression in the presence of Montelukast in Vero E6 cells).

**Figure 3-source data 1:** RT-PCR (Viral copy number in HEK ACE2 and Vero E6 cells in the presence of Montelukast).

**Figure 3-source data 2:** (Plaque assay in HEK ACE2 and Vero E6 cells in the presence of Montelukast).

**Figure 1-supplement 1-source data**: Nano-DSF screening

**Figure 1-supplement 2-source data 1**: RMSF, average hydrogen bonds.

**Figure 3- supplement 1-source data**: Toxicity assay.

**Figure 3-supplement 2 A-source data 1**: Western blot (Spike protein expression in the presence of saquinavir in HEK-ACE2 cells).

**Figure 3-supplement 2 D-source data 1**: Western blot (Spike protein expression in the presence of saquinavir in Vero E6 cells).

**Figure 3-source data 2:** RT-PCR (Viral copy number in HEK ACE2 and Vero E6 cells in the presence of saquinavir).

**Figure 3-source data 3:** (Plaque assay in HEK ACE2 and Vero E6 cells in the presence of saquinavir).
